# Vitamin D status and epigenetic-based mortality risk score: strong independent and joint prediction of all-cause mortality in a population-based cohort study

**DOI:** 10.1186/s13148-018-0515-y

**Published:** 2018-06-20

**Authors:** Xu Gao, Yan Zhang, Ben Schöttker, Hermann Brenner

**Affiliations:** 1 Division of Clinical Epidemiology and Aging Research, German Cancer Research Center (DKFZ), Im Neuenheimer Feld 581, 69120 Heidelberg, Germany; 2 Network Aging Research, University of Heidelberg, Bergheimer Straße 20, 69115 Heidelberg, Germany; 3 Division of Preventive Oncology, German Cancer Research Center (DKFZ) and National Center for Tumor Diseases (NCT), Im Neuenheimer Feld 460, 69120 Heidelberg, Germany; 4 German Cancer Consortium (DKTK), German Cancer Research Center (DKFZ), Im Neuenheimer Feld 280, 69120 Heidelberg, Germany; 5 Current Address: Department of Environmental Health Sciences, Mailman School of Public Health, Columbia University, New York, NY USA

**Keywords:** DNA methylation, Epigenetic mortality risk score, Vitamin D, All-cause mortality, Epigenetic epidemiology, Precision medicine

## Abstract

**Background:**

Vitamin D deficiency and insufficiency have been established to be strongly associated with increased overall mortality and deaths from specific aging-related diseases. Recently, an epigenetic “mortality risk score” (MS) based on whole blood DNA methylation at the 10 most prominent mortality-related cytosine-phosphate-guanine (CpG) sites has also been found to be highly related to all-cause mortality. This study aimed to explore whether vitamin D status, defined by serum 25-hydroxyvitamin D [25(OH)D] concentrations, is associated with the MS and to what extent both indicators are individually and jointly capable of predicting all-cause mortality in a general population sample of older adults.

**Results:**

The MS was derived from the blood DNA methylation profiles measured by Illumina Human Methylation 450K Beadchip, and serum 25(OH)D concentration was measured among 1467 participants aged 50–75 of the German ESTHER cohort study. There was no association between vitamin D status and the MS at baseline, but both metrics were prominently and independently associated with all-cause mortality during a median follow-up of 15.2 years. The combination of both indicators showed the potential to be a particularly strong prognostic index for all-cause mortality. Participants with vitamin D deficiency (< 30 nmol/L) and high MS (> 5 CpG sites with aberrant methylation) had almost sixfold mortality (hazard ratio 5.79, 95% CI 3.06–10.94) compared with participants with sufficient vitamin D (≥ 50 nmol/L) and a low MS (0–1 CpG site with aberrant methylation).

**Conclusions:**

This study suggests that vitamin D and the MS are strong independent predictors of all-cause mortality in older adults.

**Electronic supplementary material:**

The online version of this article (10.1186/s13148-018-0515-y) contains supplementary material, which is available to authorized users.

## Background

Vitamin D is a critical nutrient that is, apart from some limited supply from diet and supplement use, mainly obtained from the biosynthesis within the human body in response to the exposure of solar ultraviolet B radiation [1]. Vitamin D status is commonly measured via assessing 25-hydroxyvitamin D [25(OH)D] concentrations in serum [1]. Vitamin D deficiency and insufficiency have been shown to be strongly associated with increased overall mortality, as well as deaths from specific aging-related diseases, such as cardiovascular disease (CVD) and various forms of cancer [2–4]. We previously performed a meta-analysis to summarize the results of eight prospective cohort studies from European countries and the USA to investigate the prognostic association of vitamin D status and mortality [3]. Comparing bottom vs. top quintiles of 25(OH)D concentrations resulted in a risk ratio of 1.57 (95% CI 1.36–1.81) for all-cause mortality.

Recently, DNA methylation, one of the most studied and stable epigenetic modifications, has been shown to be associated with aging and aging-related health outcomes [5, 6] and recognized as the indicator for all-cause and disease-specific mortality [7]. In a recent epigenome-wide association study (EWAS) with approximately 1900 older adults followed up for 14 years and an external validation with 1727 participants, we identified 58 cytosine-phosphate-guanine (CpG) sites within 19 chromosomes that were associated with all-cause mortality [8]. We constructed a “mortality risk score” (MS) based on the 10 most robustly mortality-related loci, which was found to be a robust and informative predictor of all-cause, CVD, and cancer mortality. It is unclear, however, to what extent its association is independent of other well-established indicators of mortality risks. This study aimed to explore whether vitamin D status, defined by serum 25(OH)D concentrations, is associated with the MS and to what extent both indicators are individually and jointly capable of predicting all-cause mortality in a general population sample of older adults.

## Methods

### Study design and population

**Fig. 1:**
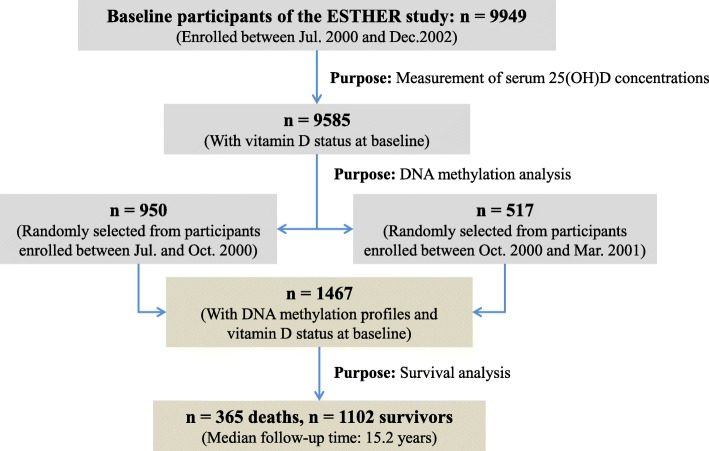
Overview of the sampling procedures of participants for analysis

Study subjects were chosen from the ESTHER study, an ongoing statewide population-based cohort study conducted in Saarland, a state located in southwestern Germany. Details of the study design have been reported previously [8, 9]. As shown in Fig. 1, 9949 older adults (aged 50–75 years) were enrolled by their general practitioners during a routine health checkup between July 2000 and December 2002 and followed up thereafter. The cross-sectional analysis of this study is based on the data and biospecimen collected at baseline from 1467 participants (close to 100% Caucasian) who were randomly selected for the measurements of 25(OH)D concentrations and DNA methylation profiles among participants recruited consecutively at the start of the ESTHER study between July 2000 and March 2001 [10]. Participants were then regularly followed up with respect to the incidence of major chronic diseases and mortality. The ESTHER study was approved by the ethics committees of the University of Heidelberg and the state medical board of Saarland, Germany. Written informed consent was obtained from all participants.

### Vitamin D measurements

Blood samples were taken during the health checkup and stored at − 80 °C until further processing. As previously described [11], the automated Diasorin–Liaison analyzer (Diasorin, Inc.) was used to measure total serum 25(OH)D concentrations in women from baseline serum samples in the central laboratory of the University Clinic of Heidelberg in 2006 within the framework of a project on women’s health. Additional funding was obtained in 2009 to measure total serum 25(OH)D concentrations in men as well. As the Diasorin–Liaison method used for women was no longer available at that time, the automated IDS-iSYS analyzer (Immunodiagnostic Systems, GmbH) was used instead. Both assays with their within- and between-assay coefficients and lower limits of detection have been comprehensively described elsewhere [12, 13]. Both immunoassays were standardized retrospectively to measurements by liquid chromatography-tandem mass spectrometry (LC-MS/MS), the gold standard method [14], in a random subsample of 97 males and 97 females as previously described [12, 13]. Vitamin D status defined by total serum 25(OH)D concentrations were classified into three categories with the following criteria: deficiency, < 30 nmol/L; insufficiency, 30 to < 50 nmol/L; and sufficiency, ≥ 50 nmol/L.

### DNA methylation data

DNA from whole blood samples was collected using a salting out procedure [15]. DNA methylation profiles were determined by the Illumina Human Methylation 450K Beadchip (Illumina, San Diego, CA, USA). As previously described [10], samples were analyzed following the manufacturer’s instruction at the Genomics and Proteomics Core Facility of the German Cancer Research Center, Heidelberg, Germany. Illumina’s GenomeStudio® (version 2011.1; Illumina Inc.) was employed to extract DNA methylation signals from the scanned arrays (Module version 1.9.0; Illumina Inc.). The methylation level of a specific CpG site was quantified as a *β* value ranging from 0 (no methylation) to 1 (full methylation). According to the manufacturer’s protocol, no background correction was done and data were normalized to internal controls provided by the manufacturer. All controls were checked for inconsistencies in each measured plate. Probes with a detection *p* value > 0.05 were excluded from analysis. Illumina normalization and preprocessing methods implemented in Illumina’s GenomeStudio® were utilized.

As described by Zhang et al., 10 CpG sites (cg01612140, cg05575921, cg06126421, cg08362785, cg10321156, cg14975410, cg19572487, cg23665802, cg24704287, and cg25983901) were selected from the whole epigenome data to build the MS [8]. Values within the fourth quartile of cg08362785 and first quartile of the other nine loci were defined to reflect aberrant methylation for each CpG site, and the ordinal MS was determined as the cumulative number of aberrantly methylated CpG sites (0–10). The participants were further classified into three risk levels: low: MS = 0–1, moderate: MS = 2–5, and high: MS > 5.

### Covariate and outcome assessment

Information on socio-demographic characteristics, lifestyle factors, dietary habits, and health status at baseline was obtained by standardized self-administered questionnaires [16]. Participants were asked about their past and present smoking behaviors and were then categorized into current, former, and never smokers. Information on BMI and systolic blood pressure was extracted from a standardized form filled by the general practitioners during the health checkups. Serum total cholesterol and C-reactive protein (CRP) were measured with Backman Synchon LX and turbudimetry, respectively [11]. Prevalent CVD at baseline was defined by either physician-reported coronary heart disease or a self-reported history of a major cardiovascular event, such as myocardial infarction, stroke, pulmonary embolism, or revascularization of coronary arteries. Prevalent diabetes was defined by physician diagnosis or the use of glucose-lowering drugs. Prevalent cancer [ICD-10 C00-C99 except non-melanoma skin cancer (C44)] was determined by self-report or record linkage with data from the Saarland Cancer Registry (in German) [17].

Deaths of follow-up until the end of 2015 were retrieved by record linkage with population registries in Saarland. Participants who moved out of Saarland were considered as censored at the date last known to be alive. Information on the causes of death was obtained from death certificates provided by local public health offices and was coded with ICD-10 codes.

### Statistical analysis

First, major socio-demographic characteristics, lifestyle factors, and the MS at baseline of 1467 participants, overall and stratified by vitamin D status, were summarized by descriptive statistics, and differences among subsets were tested for statistical significance by Kruskal-Wallis test (continuous variables) and chi-square test (categorical variables).

We then examined the associations of vitamin D status and MS (risk levels) with all-cause mortality using three multivariate Cox regression models which increasingly adjusted for potential covariates, including age (years), sex (male/ female), smoking status (current/former/never smoking), alcohol consumption (g/day), BMI class [kg/m^2^; underweight (< 18.5, < 1% of the study population) or normal weight (18.5 to < 25), overweight (25 to < 30), obese (≥ 30)], physical activity [inactive (< 1 h of physical activity/week), medium or high (≥ 2 h of light or ≥ 2 h of vigorous physical activity/week), low (other)], the prevalence of CVD (yes/no), diabetes (yes/no) and cancer (yes/no), systolic blood pressure (mmHg), CRP (mg/L), total cholesterol (mg/dL), intake of vitamin supplements (yes/no), fish consumption (≤ 1 time/week/> 1 time/week), and the season of blood draw (spring: March–May; summer: June–August; autumn: September–November; winter: December–February). Leukocyte distribution estimated by Houseman’s algorithm was additionally controlled for in the models in which the MS was involved [18]. The dose-response curves of continuous 25(OH)D concentrations and ordinal MS with all-cause mortality were evaluated by restricted cubic spline regression using the SAS macro from Desquilbet et al. [19]. All models for dose-response analyses were adjusted for the covariates described above. The 25th, 50th, and 75th percentiles of the MS and the 30, 50, and 70 nmol/L of serum 25(OH)D concentrations were selected as knots for each marker, respectively. In addition to models including either vitamin D status or MS (risk levels) as the predictor, we further evaluated their independent associations with all-cause mortality in a model containing both predictors.

Finally, we assessed the joint associations of both indicators with all-cause mortality. Survival was first compared by the health status defined by the combination of both indicators using direct-adjusted survival curves (adjusted for age and sex), followed by Cox regression analyses with multivariable adjustment. In addition, adjusted survival curves were constructed and Cox models were run to assess the associations of MS with mortality within subgroups of participants defined by vitamin D status and of vitamin D status with mortality within subgroups of participants defined by the MS (risk levels). To avoid overadjustment, only covariates related to either of the two predictors (*p* value < 0.2 in bivariable analyses) were included in the models assessing joint associations.

All analyses were performed by SAS version 9.4 (SAS Institute Inc., Cary, NC, USA). For all statistical analyses, a *p* value less than 0.05 in two-sided tests was considered as statistically significant.

## Results

### Participant characteristics

Characteristics of all 1467 participants and the subsets based on vitamin D status are shown in Table 1. For the total population, the average age was about 62 years. More than half of the participants were smokers (current or former smokers). The majority of participants were overweight or obese, reported no or low amounts of alcohol consumption, and no or only low physical activity. More than half of the participants had a MS > 1, and about 46% had a MS of 2–5. The mean standardized serum 25(OH)D concentration was 52.1 nmol/L, with 15.2 and 43.4% of participants meeting the criteria for vitamin D deficiency (< 30 nmol/L) and insufficiency (30 to < 50 nmol/L), respectively. During a median follow-up time of 15.2 years, 365 (24.9%), 128 (8.9%), and 134 (9.3%) participants died from any cause, CVD, or cancer, respectively.

**Table 1:** Characteristics of the ESTHER study participants at baseline according to vitamin D status

Characteristics	Total population	Subsets based on vitamin D status			
		< 30 nmol/L/deficiency	30 to < 50 nmol/L/insufficiency	≥ 50 nmol/L/sufficiency	*p* value
*N*	1467	223 (15.2%)	637 (43.4%)	607 (41.4%)	
Age (years)	62.1 (6.51)	62.4 (6.91)	62.4 (6.43)	61.6 (6.42)	0.105
Sex (male)	663 (45.2%)	61 (27.4%)	216 (33.9%)	386 (63.6%)	< 0.0001
Smoking status					< 0.0001
Current smoker	271 (18.5%)	46 (20.6%)	117 (18.4%)	108 (17.8%)	
Former smoker	488 (33.3%)	61 (27.4%)	172 (27.0%)	255 (42.0%)	
Never smoker	708 (48.3%)	116 (52.0%)	348 (54.6%)	244 (40.2%)	
Body mass index ^a^					0.0006
Underweight or normal weight (< 25.0)	397 (27.1%)	64 (28.7%)	161 (25.3%)	172 (28.3%)	
Overweight (25 to < 30)	677 (46.2%)	91 (40.8%)	278 (43.6%)	308 (50.7%)	
Obese (≥ 30.0)	390 (26.6%)	68 (30.5%)	196 (30.8%)	126 (20.8%)	
Alcohol consumption ^b^					< 0.0001
Abstainer	466 (31.8%)	92 (41.3%)	222 (34.9%)	152 (25.0%)	
Low	797 (54.3%)	100 (44.8%)	319 (50.1%)	378 (62.3%)	
Intermediate	78 (5.3%)	5 (2.2%)	29 (4.6%)	44 (7.3%)	
High	20 (1.4%)	2 (0.9%)	13 (2.0%)	5 (0.8%)	
Physical activity ^c^					< 0.0001
Inactive	293 (20.0%)	51 (22.9%)	150 (23.6%)	92 (15.2%)	
Low	673 (45.9%)	114 (51.1%)	295 (46.3%)	264 (43.5%)	
Medium or high	501 (34.2%)	58 (26.0%)	192 (30.1%)	251 (41.4%)	
Prevalence of major diseases					
Cardiovascular disease	309 (21.1%)	46 (20.6%)	126 (19.8%)	137 (22.6%)	0.476
Diabetes ^d^	228 (15.5%)	41 (18.4%)	100 (15.7%)	87 (14.3%)	0.352
Cancer	87 (5.9%)	15 (6.7%)	40 (6.3%)	32 (5.3%)	0.649
Systolic blood pressure (mmHg) ^e^	139.9 (19.79)	140.8 (21.01)	140.2 (19.69)	139.4 (19.46)	0.537
C-reactive protein (mg/L) ^f^	3.7 (7.28)	3.4 (4.42)	3.6 (8.23)	3.9 (7.07)	0.979
Total cholesterol (mg/dL) ^g^	183.5 (60.23)	179.2 (66.15)	178.1 (59.23)	190.7 (58.3)	0.0002
Intake of vitamin D supplements (yes) ^h^	563 (38.4%)	83 (37.2%)	244 (38.3%)	236 (38.9%)	0.934
Fish consumption (> 1 time/week) ^i^	925 (63.1%)	133 (59.6%)	403 (63.3%)	389 (64.1%)	0.740
Mortality risk score (risk levels)					0.872
0–1/low	602 (41.0%)	96 (43.1%)	263 (41.3%)	243 (40.0%)	
2–5/moderate	670 (45.7%)	95 (42.6%)	292 (45.8%)	283 (46.6%)	
> 5/high	195 (13.3%)	32 (14.3%)	82 (12.9%)	81 (13.3%)	

Mean values (standard deviation) for continuous variables and *n* (%) for categorical variables. Differences among subgroups of vitamin D status were tested by Kruskal-Wallis test (continuous variables) and chi-square test (categorical variables)

^a^Data missing for three participants

^b^Data missing for 106 participants. Categories are defined as follows: abstainer, low (women, 0 to < 20 g/day; men, 0 to < 40 g/day), intermediate (20 to < 40 g/day and 40 to < 60 g/day, respectively), high (≥ 40 and ≥ 60 g/day, respectively)

^c^Categories are defined as follows: inactive (< 1 h of physical activity/week), medium or high (≥ 2 h of vigorous or ≥ 2 h of light physical activity/week), low (other)

^d^Data missing for 16 participants

^e^Data missing for 27 participants

^f^Data missing for 30 participants

^g^Data missing for two participants

^h^Data missing for 62 participants

^i^Data missing for 56 participants

Participants with sufficient vitamin D (≥ 50 nmol/L) included larger proportions of males, former smokers, consumers of low amounts of alcohol, were less often obese and more often reported medium or high physical activity than those with vitamin D deficiency and insufficiency. By contrast, no differences in the prevalence of major diseases and in the distribution of the MS were observed among participants with vitamin D deficiency, insufficiency, or sufficiency.

### Individual associations of vitamin D status and the mortality risk score with all-cause mortality

Table 2 shows the associations of vitamin D status and MS (risk levels) with all-cause mortality among 1467 participant during a median follow-up time of 15.2 years. Significant associations were observed between each indicator and mortality, which persisted after controlling for multiple covariates. Vitamin D insufficiency and deficiency were associated with about 1.5- [hazard ratio (HR) 1.46; 95% CI 1.11–1.93] and 2.0 (HR 1.99; 95% CI 1.38–2.87)-fold mortality compared to the vitamin D sufficiency subgroup after adjustment of multiple covariates (model 3). The moderate and high MS risk levels were associated with about 1.9- (HR 1.87; 95% CI 1.37–2.54) and 3.4 (HR 3.42; 95% CI 2.32–5.04)-fold mortality compared to the low MS risk level. Additional mutual adjustment for both indicators did not alter the patterns in any relevant manner with essentially unchanged HRs.

**Table 2:** Associations of vitamin D status and mortality risk score (risk levels) with all-cause mortality

Characteristics	*N*_total_	*N*_death_	Model 1 ^a^		Model 2 ^b^		Model 3 ^c^		Model with both indicators ^d^	
			HR (95% CI)	*p* value	HR (95% CI)	*p* value	HR (95% CI)	*p* value	HR (95% CI)	*p* value
Vitamin D status										
≥ 50 nmol/L /sufficiency	607	138	Reference		Reference		Reference		Reference	
30 to < 50 nmol/L/insufficiency	637	156	1.31 (1.03–1.67)	0.027	1.30 (1.01–1.69)	0.045	1.46 (1.11–1.93)	0.008	1.43 (1.08–1.88)	0.013
< 30 nmol/L/deficiency	223	71	2.03 (1.48–2.79)	< 0.0001	1.95 (1.39–2.74)	0.0001	1.99 (1.38–2.87)	0.0003	2.08 (1.44–3.01)	< 0.0001
Mortality risk score (risk levels)										
0–1/low	602	86	Reference		Reference		Reference		Reference	
2–5/moderate	670	180	1.85 (1.42–2.41)	< 0.0001	1.76 (1.33–2.33)	< 0.0001	1.87 (1.37–2.54)	< 0.0001	1.89 (1.39–2.57)	< 0.0001
> 5/high	195	99	4.13 (3.04–5.62)	< 0.0001	2.99 (2.11–4.24)	< 0.0001	3.42 (2.32–5.04)	< 0.0001	3.38 (2.30–4.96)	< 0.0001

*HR* hazard ratio, *CI* confidence interval

^a^Adjusted for age, sex, and the season of blood draw; the leukocyte distribution (Houseman algorithm) was additionally adjusted for the models with mortality risk score

^b^Additionally adjusted for alcohol consumption, smoking status, BMI class, physical activity, regular intake of vitamin supplements, and fish consumption

^c^Additionally adjusted for the prevalence of CVD, diabetes and cancer, systolic blood pressure, CRP, and total cholesterol

^d^Adjusted for the covariates added in model 3

**Fig. 2:**
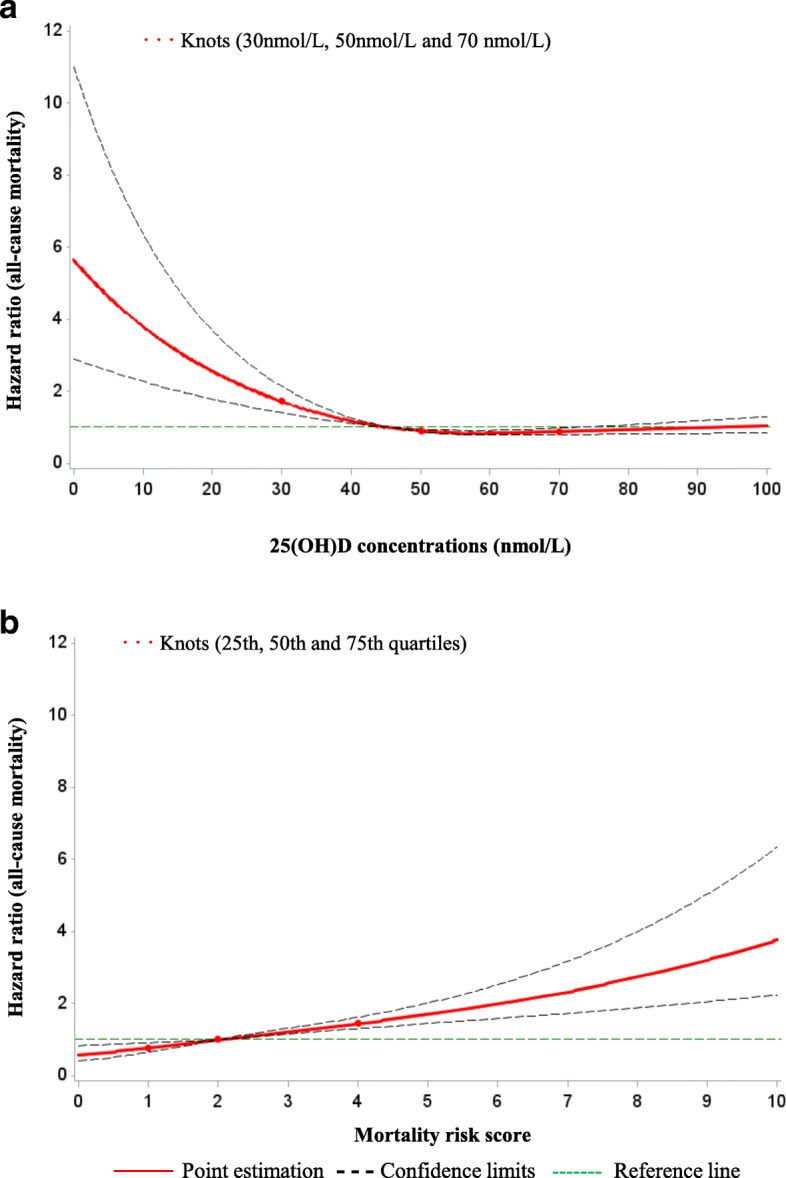
Graphs of the best-fitting models for relationships of 25(OH)D concentrations (**a**) and the ordinal mortality risk score (**b**) with all-cause mortality. Legend: red lines, estimation; dashed lines, confidence limits; red dots, knots; green lines, reference lines

Figure 2 further depicts the dose-response relationships of 25(OH)D concentrations and ordinal MS with all-cause mortality after controlling for all potential covariates. Mortality strongly increased for 25(OH)D concentrations < 40 nmol/L and steadily increased with increasing MS.

### Joint associations of vitamin D status and mortality risk score with all-cause mortality

**Fig. 3:**
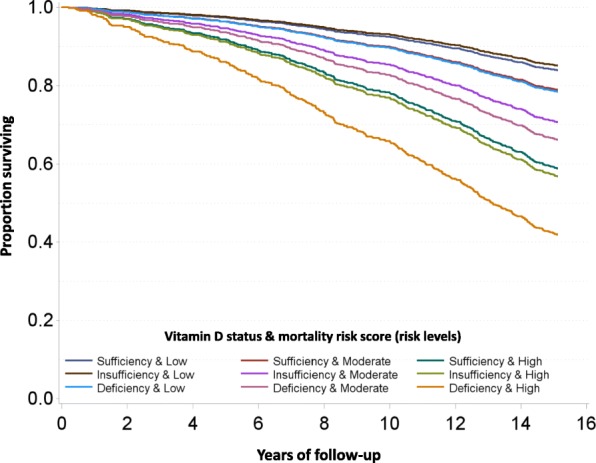
Age- and sex-adjusted survival curves for the joint associations of vitamin D status and mortality risk score (risk levels) with all-cause mortality (log-rank *p* value < 0.0001). Legend: Each curve represents the subgroup defined by the combination of vitamin D status and mortality risk score (risk levels)

Figure 3 presents the direct-adjusted survival curves for the joint association of vitamin D status and the MS (risk levels) with all-cause mortality during a median follow-up time of 15.2 years. People in the highest risk group with vitamin D deficiency and high MS risk level had a substantially higher overall death rate than all other groups. After adjustment for the selected covariates based on the results of bivariate associations (Additional file 1: Table S1), a clearly increasing mortality risk (i.e., HR) was observed with both vitamin D deficiency and a high MS (Table 3). In particular, people in the highest risk group of both factors had about 5.8-fold mortality compared to the people in the reference group that had sufficient vitamin D and a low MS (HR 5.79; 95% CI 3.06–10.94).

**Table 3:** Joint associations of vitamin D status and mortality risk score (risk levels) with all-cause mortality: joint association using people with sufficient vitamin D and low mortality risk score as reference group

Characteristics	Mortality risk score (risk levels)								
	0–1/low			2–5/moderate			> 5/high		
	Death/total	HR (95% CI)	*p* value	Death/total	HR (95% CI)	*p* value	Death/total	HR (95% CI)	*p* value
Vitamin D status (category)									
≥ 50 nmol/L/sufficiency	34/243	Reference		65/283	1.28 (0.81–2.03)	0.292	39/81	2.50 (1.48–4.22)	0.0006
30 to < 50 nmol/L/insufficiency	34/263	0.96 (0.56–1.65)	0.894	83/292	2.21 (1.41–3.45)	0.0005	39/82	3.34 (1.94–5.75)	< 0.0001
< 30 nmol/L/deficiency	18/96	1.50 (0.79–2.86)	0.214	32/95	2.86 (1.65–4.97)	0.0002	21/32	5.79 (3.06–10.94)	< 0.0001

*HR* hazard ratio, *CI* confidence interval

After the selection of covariates based on bivariate associations, models are adjusted for age, sex, BMI class, smoking status, physical activity, alcohol consumption, CRP, total cholesterol, the prevalence of cardiovascular disease and diabetes, season of blood draw, and the selected leukocyte distribution (Houseman algorithm, including CD4+ T cells, CD8+ T cells, B cells, and granulocytes)

We further stratified the total population by each indicator and assessed the association of the other indicator with all-cause mortality. As demonstrated in Tables 4 and 5 and Additional file 2: Figure S1, the identified patterns in subgroup-specific analyses are in line with the findings for the total study population. However, the association of a high MS with all-cause mortality was particularly strong among participants with vitamin D deficiency (HR 6.90; 95% CI 2.83–16.83). Additional sex-specific analysis yielded similar patterns for both sexes (data not shown).

**Table 4:** Joint associations of vitamin D status and mortality risk score (risk levels) with all-cause mortality: associations of mortality risk score with all-cause mortality within subgroups defined by vitamin D status

Characteristics	Mortality risk score (risk levels)								
	0–1/low			2–5/moderate			> 5/high		
	Death/total	HR (95% CI)	*p* value	Death/total	HR (95% CI)	*p* value	Death/total	HR (95% CI)	*p* value
Vitamin D status (category)									
Overall	86/602	Reference		180/670	1.77 (1.32–2.36)	0.0001	99/195	3.24 (2.24–4.68)	< 0.0001
≥ 50 nmol/L/sufficiency	34/243	Reference		65/283	1.18 (0.73–1.91)	0.504	39/81	2.44 (1.34–4.43)	0.003
30 to < 50 nmol/L/insufficiency	34/263	Reference		83/292	2.62 (1.65–4.15)	< 0.0001	39/82	4.37 (2.37–8.05)	< 0.0001
< 30 nmol/L/deficiency	18/96	Reference		32/95	2.21 (1.11–4.38)	0.024	21/32	6.90 (2.83–16.83)	< 0.0001

*HR* hazard ratio, *CI* confidence interval

After the selection of covariates based on bivariate associations, models are adjusted for age, sex, BMI class, smoking status, physical activity, alcohol consumption, CRP, total cholesterol, the prevalence of cardiovascular disease and diabetes, season of blood draw, and the selected leukocyte distribution (Houseman algorithm, including CD4+ T cells, CD8+ T cells, B cells, and granulocytes)

**Table 5:** Joint associations of vitamin D status and mortality risk score (risk levels) with all-cause mortality: associations of vitamin D status with all-cause mortality within subgroups defined by mortality risk score

Characteristics	Mortality risk score (risk levels)											
	Overall			0–1/low			2–5/moderate			> 5/high		
	Death/total	HR (95% CI)	*p* value	Death/total	HR (95% CI)	*p* value	Death/total	HR (95% CI)	*p* value	Death/total	HR (95% CI)	*p* value
Vitamin D status (category)												
≥ 50 nmol/L/sufficiency	138/607	Reference		34/243	Reference		65/283	Reference		39/81	Reference	
30 to < 50 nmol/L/insufficiency	156/637	1.43 (1.10–1.86)	0.007	34/263	0.91 (0.51–1.64)	0.758	83/292	1.69 (1.16–2.48)	0.007	39/82	1.30 (0.79–2.17)	0.295
< 30 nmol/L/deficiency	71/223	2.01 (1.41–2.85)	< 0.0001	18/96	1.64 (0.78–3.45)	0.193	32/95	2.16 (1.30–3.60)	0.003	21/32	1.97 (1.01–3.84)	0.047

*HR* hazard ratio, *CI* confidence interval

After the selection of covariates based on bivariate associations, models are adjusted for age, sex, BMI class, smoking status, physical activity, alcohol consumption, CRP, total cholesterol, the prevalence of cardiovascular disease and diabetes, season of blood draw, and the selected leukocyte distribution (Houseman algorithm, including CD4+ T cells, CD8+ T cells, B cells, and granulocytes)

## Discussion

In this study with 1467 older adults recruited in a population-based cohort with both measurements of serum 25(OH)D concentrations and DNA methylation profiles at baseline, vitamin D status and an epigenetic-based MS strongly and independently predicted mortality from any causes. In particular, the combination of both indicators demonstrated the potential to be a robust prognostic marker for all-cause mortality with an approximately sixfold mortality among those with vitamin D deficiency and a high MS compared to participants with sufficient vitamin D and a low MS.

Previous studies suggested that there might be a reciprocal relationship between vitamin D and epigenetic changes: epigenetic alterations regulate the expression of vitamin D receptor genes and vitamin D could influence epigenetic events [20]. Previous studies have also suggested epigenetic effects of vitamin D on histone modification, another form of epigenetic regulation [20, 21]. Although a potential link between severe vitamin D deficiency and DNA methylation has been reported in African American adolescents [22], no such link was observed in a previous analysis of the ESTHER study [23]. In addition, Chavez et al. using the Illumina 450K array did not find significant alterations in DNA methylation profiles when they exposed human blood cells to vitamin D for up to 120 h [24]. In our study, vitamin D deficiency was not related to the methylation of the sites included in the MS. Among the 10 CpG sites of the MS, four loci are smoking-related, including cg05575921 (*AHRR*), cg06126421 (*6p21.33*), cg19572487 (*RARA*), and cg01612140 (*6q14.1*) [25]. Even though vitamin D sufficiency may have a protective effect against the damaging effects of smoking on lung function [26], we did not observe any association of the MS and vitamin D status. Along the same lines, although loci cg08362785 and cg23665802 are mapped to genes *MKL1* and *MIR19A* that are associated with the risks of lung and breast cancer [27, 28], vitamin D, which was found to be related to the incidence and mortality of both cancers [29], did not show any relation with either locus nor the MS.

Associations between both vitamin D deficiency and the MS, and all-cause mortality have been identified and validated by previous studies among older adults [3, 7, 8, 30]. To our best knowledge, this study is the first investigation which further demonstrated that both markers are not only capable of independently predicting the death from any cause over 15 years of follow-up, but also can be integrated together to be an extraordinarily strong indicator for mortality.

In particular, the combination of vitamin D and the MS appears to be a better predictor of all-cause mortality than any previously suggested biomarkers of mortality risk [31]. The strength of the association is the more remarkable, as most of the deaths in our study with long-term follow-up occurred many years after baseline measurements of vitamin D and MS, whereas the strength of prediction for most biomarkers is typically attenuated with increasing length of follow-up [32, 33]. Even stronger associations of both biomarkers and their combinations with mortality were seen when the follow-up was restricted to 5 or 10 years, even though confidence intervals became wider due to the lower numbers of deaths (data not shown).

One of the major strengths of this study is the availability of a broad range of covariates adjusted for in addition to epigenome-wide methylation data in a population-based cohort that was comprehensively followed up with high completeness over 15 years. We also acknowledge several limitations in the interpretation of results. First, the overall number of deaths for the survival analyses was limited, which caused rather wide confidence intervals for some of the HR estimates. Furthermore, shifts of leukocyte distribution might affect the associations of DNA methylation in whole blood samples [34]. Hence, we adjusted for leukocyte distribution by the Houseman algorithm to restrict potential confounding from differential blood counts to the greatest possible extent [18]. In addition, 25(OH)D concentrations were originally measured by two different methods. Nevertheless, standardization by the gold standard LC-MS/MS method, which yielded results that were highly correlated with original measurements [13], should ensure comparability and validity of the vitamin D status included in our analysis. Finally, due to the heterogeneity of populations with respect to food fortification, use of dietary supplements, UV radiation levels, and susceptibility of the epigenome in response to external exposure, further studies are needed to evaluate to what extent our findings can be generalized to other populations: in particular younger individuals and non-Caucasians.

## Conclusions

In summary, our results contribute to a rapidly growing body of literature investigating the prediction of age-related morbidity and long-term mortality by various markers identified from environmental, genetic, and epigenetic research. We showed that the application of both vitamin D status and the MS based on DNA methylation signatures yielded a robust, independent predictor for all-cause mortality, suggesting that vitamin D status and DNA methylation signatures in combination may be most useful in risk stratification as potential intermediate biomarkers. Further studies are warranted to elucidate the underlying pathophysiological mechanisms and potential clinical applications of the combination of both indicators in routine medical practice and intervention research aimed at reducing mortality.

## Additional files

Additional file 1: **Table S1.** Bivariate associations of vitamin D status and mortality risk score (risk levels) with potential confounders. Legend: Each curve represents the subgroup defined by the combination of vitamin D status and mortality risk score (risk levels). (DOCX 16 kb)

Additional file 2: **Figure S1.** Age and sex-adjusted survival curves for joint associations of mortality risk score/vitamin D status with all-cause mortality within subgroups defined by vitamin D status/mortality risk score (all log-rank *p* values < 0.05). (PDF 428 kb)

## Abbreviations

25(OH)D: 25-Hydroxyvitamin D
BMI: Body mass index
CI: Confidence interval
CpG: Cytosine-phosphate-guanine
HR: Hazard ratio
MS: Mortality risk score
