# Supplementary material for: Vitamin D status and epigenetic-based mortality risk score: strong independent and joint prediction of all-cause mortality in a population-based cohort study
Source: Clin Epigenetics. 2018 Jun 20;10:84. doi: 10.1186/s13148-018-0515-y (PMC6011585; doi:10.1186/s13148-018-0515-y)
Supplement: Supplementary file 1 — Table S1. Bivariate associations of vitamin D status and mortality risk score (risk levels) with potential confounders. Legend: Each curve represents the subgroup defined by the combination of vitamin D status and mortality risk score (risk levels). (DOCX 16 kb) [file 13148_2018_515_MOESM1_ESM.docx]

**Table S1** Bivariate associations of vitamin D status and mortality risk score (risk levels) with potential confounders ^a^

| **Potential confounders** | **Vitamin D status** | **Mortality risk score**  **(risk levels)** | **Combination** | **Selected** |
| --- | --- | --- | --- | --- |
|  | ***p*-value** | ***p*-value** | ***p*-value** |  |
| Physical activity | <0.0001 | 0.128 | <0.0001 | √ |
| Prevalence of diabetes | 0.354 | 0.023 | 0.0595 | √ |
| Prevalence of C-reactive protein | 0.460 | <0.0001 | <0.0001 | √ |
| Prevalence of cancer | 0.723 | 0.092 | 0.6326 |  |
| Regular intake of vitamin supplements | 0.913 | 0.054 | 0.3892 |  |
| Fish intake | 0.739 | 0.473 | 0.495 |  |
| Season of blood draw | <0.0001 | 0.042 | <0.0001 | √ |
| Alcohol consumption | <0.0001 | 0.2843 | <0.0001 | √ |
| Total cholesterol | 0.0002 | 0.1166 | 0.0014 | √ |
| C-reactive protein | 0.981 | <0.0001 | <0.0001 | √ |
| Systolic blood pressure | 0.492 | 0.3378 | 0.7886 |  |
| NK cells | 0.585 | 0.0491 | 0.2289 |  |
| CD4+ T-cells | 0.775 | 0.0056 | 0.0297 | √ |
| CD8+ T-cells | 0.344 | 0.0011 | 0.0248 | √ |
| B-cells | 0.912 | 0.0022 | 0.0199 | √ |
| Monocytes | 0.519 | 0.7008 | 0.4429 |  |
| Granulocytes | 0.772 | 0.0001 | 0.0013 | √ |

a: Covariates with *p*-values < 0.2 were selected as covariates in the joint and marginal model; the categorical variables were tested by Chi-Square test and the continuous variables were tested by Kruskal-Wallis Test;
