# Supplementary material for: Vitamin D status and epigenetic-based mortality risk score: strong independent and joint prediction of all-cause mortality in a population-based cohort study
Source: Clin Epigenetics. 2018 Jun 20;10:84. doi: 10.1186/s13148-018-0515-y (PMC6011585; doi:10.1186/s13148-018-0515-y)
Supplement: Supplementary file 2 — Figure S1. Age and sex-adjusted survival curves for joint associations of mortality risk score/vitamin D status with all-cause mortality within subgroups defined by vitamin D status/mortality risk score (all log-rank p values < 0.05). (PDF 428 kb) [file 13148_2018_515_MOESM2_ESM.pdf]

**a) Associations of mortality risk score with all-cause mortality within subgroups defined by vitamin D status**

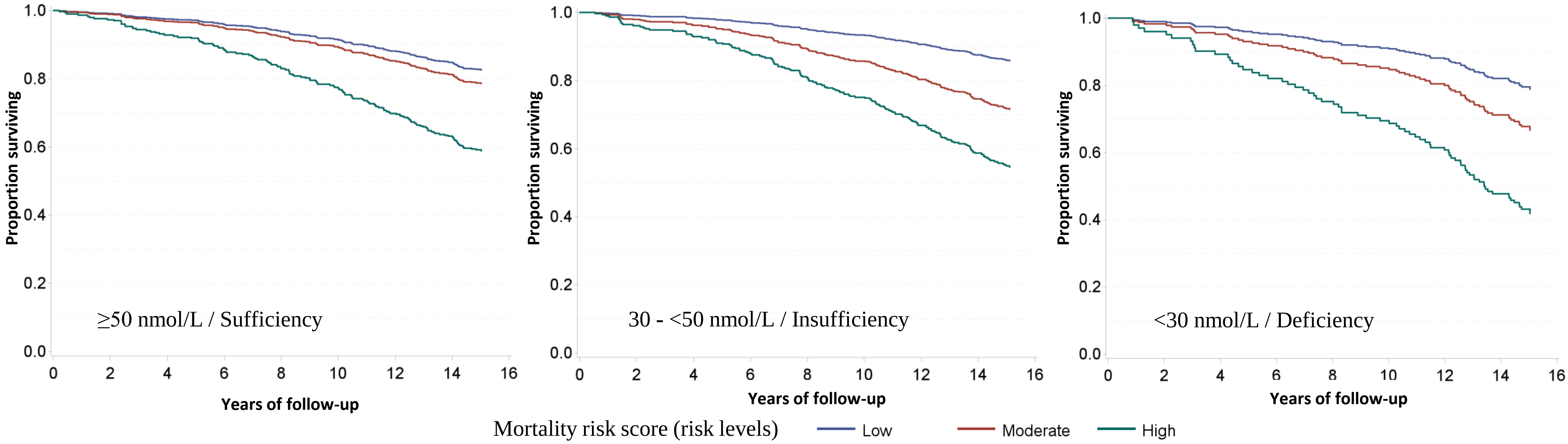


**b) Associations of vitamin D status with all-cause mortality within subgroups defined by mortality risk score**

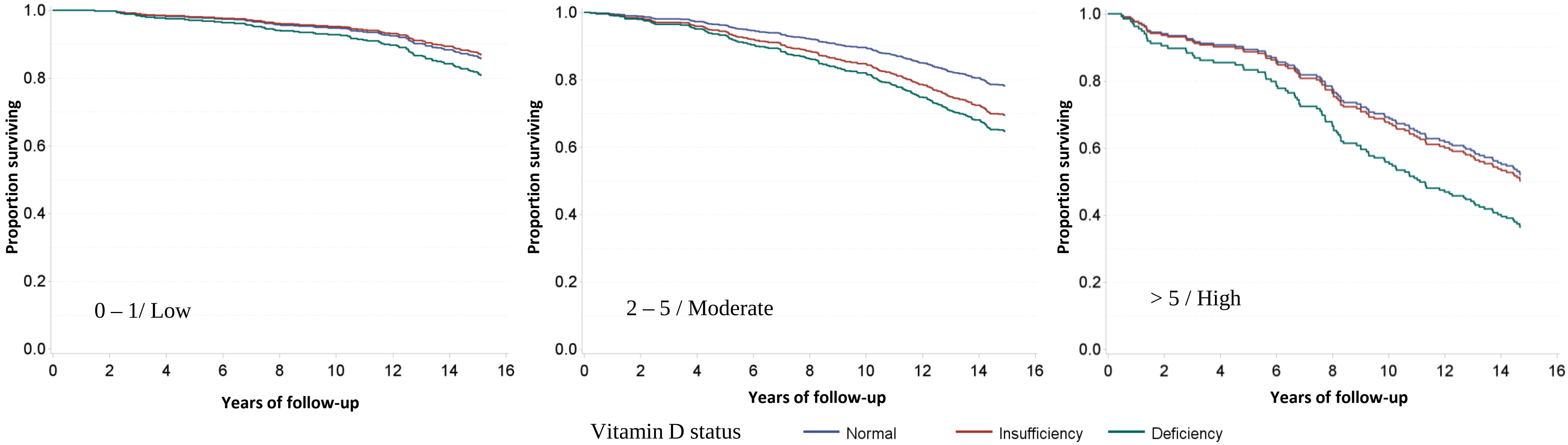


**Figure S1** Age and sex adjusted survival curves for joint associations of mortality risk score/ vitamin D status with all-cause mortality within subgroups defined by vitamin D status/ mortality risk score (all log-rank p-values <0.05)
